# Improving Health Care with Advanced Analytics: Practical Considerations

**DOI:** 10.5334/egems.276

**Published:** 2019-03-25

**Authors:** Jose Benuzillo, Lucy A. Savitz, Scott Evans

**Affiliations:** 1 Kaiser Permanente, US; 2 Intermountain Healthcare, US

**Keywords:** Advanced analytics, machine learning, predictive analytics, artificial intelligence, analytic tools

## Abstract

Artificial intelligence (AI) is becoming ubiquitous in health care, largely through machine learning and predictive analytics applications. Recent applications of AI to common health care scenarios, such as screening and diagnosing, have fueled optimism about the use of advanced analytics to improve care. Careful and objective considerations need to be made before implementing an advanced analytics solution. Critical evaluation before, during, and after its implementation will ensure safe care, good outcomes, and the elimination of waste. In this commentary we offer basic practical considerations for developing, implementing, and evaluating such solutions based on many years of experience.

## Introduction

Artificial intelligence (AI) is becoming ubiquitous in health care, largely through machine learning and predictive analytics applications. Although many of these tools have been available for decades, their recent application to common health care scenarios, such as screening and diagnosing, has fueled optimism about the use of advanced analytics to improve care. For example, researchers recently demonstrated that a deep convolutional neural network (CNN) may enable automated screening and diagnosis for retinopathy of prematurity with high accuracy and repeatability. Their algorithm diagnosed 91 of 100 images (91.0 percent) correctly, whereas 8 experts had an average accuracy of 82.0 percent [1]. A CNN also outperformed the majority of 58 dermatologists tested in accurately diagnosing melanoma, with a median area under the receiver operating characteristic curve of 0.86 compared with 0.79, P < 0.01 [2]. Similarly, researchers from Johns Hopkins University developed a novel machine-learning approach that provides rapid, remote, frequent, and objective assessment of Parkinson’s disease symptom severity using smartphones [3]. These tools are important contributors to consistent decision quality in treatment planning.

Perhaps the most common example of advanced analytics in health care, however, is predictive models to identify high-risk patients [4,5,6,7,8]. Potential benefits of these analytic tools include the early identification of patients at high-risk for 30-day readmission or mortality. In addition, the knowledge derived from these predictive models holds promise as a tool to target limited resources and address pressing public health issues such as preventing suicide attempts in adolescents [9].

Without a doubt, advanced analytics is transforming health care at a rapid pace. This transformation has been propelled by advances in computational power; the spreading of algorithms capable of simplifying complex tasks; the increasing availability of large amounts of data; and the number and diversity of innovators in this area. Advanced analytics are used in domains ranging from electronic health records to imaging and diagnostics, remote monitoring, drug discovery, billing and fraud prevention, and molecular profiling.

## Overoptimism or a critical tool for improving health care?

The potential benefits of applying advanced analytics in health care seem to be indisputable. However, evidence that these advanced analytics tools improve care and outcomes in a cost-effective, measurable way is limited. Many of the advanced analytics articles focus on the methods used, the assumptions made, the validation efforts undertaken, and the limitations. Few efforts have been made to include details about real-world performance of analytic tools, the clinical interventions that accompanied computer-generated knowledge, the implementation strategy adopted, the disruption to existing workflows, the adoption of technology, the education provided to both clinicians and patients about the use of advanced analytics in their care interaction, or cost-effectiveness. The promise of advanced analytics in health care is tremendous; however, ignorance of these challenges may overshadow its potential clinical impact and could result in waste. In this article, we offer basic practical considerations to individuals in health care settings who are involved in developing, buying, piloting, implementing, and evaluating advanced analytics solutions.

## Considerations before selecting an advanced analytics solution

A comprehensive list of factors to consider is outside the scope of this commentary, but we offer some basic requirements based on many years of experience developing, implementing, and evaluating advanced analytics tools:

1. **Define a use case:** Documenting the functional requirements of the advanced analytics solution is an important and valuable requirement for developing a solution. This is the time to ask how critical addressing a problem with an advanced analytics solution is to the organization? Would a tool improve care by making it safer or cheaper? How difficult would the solution be to implement and scale? This is an opportunity to clearly define what the solution will and won’t do and who needs to be involved. Besides listing the inputs and the outputs and the clinical workflows involved, a consideration of how this advanced analytics solution will fit with the broader health care ecosystem should be made. It is also the time to assess the existing infrastructure and the capacity and capabilities of the organization.
2. **Decide whether to build or buy:** While building offers the greatest flexibility, the long-term maintenance costs are usually overlooked or minimized. Individuals with the skillset to develop advanced analytics are highly specialized. Sometimes, the development and maintenance of the analytic solution depends on a small team of talented individuals that may end up leaving the company without a transition plan. Our experience is that a significant investment of time and resources is necessary to build a solution. In addition, when the health care system builds the analytic solution, it assumes the liability for data security and its correct performance. In contrast, buying offers little or no flexibility to customization but mitigates many of the challenges mentioned above. As part of the decision making to buy an “off-the-shelf” solution, ask for a list of similar organizations who purchased the solution and make an effort to investigate their experiences with the product in question and with the technical and customer support teams. The cost of advanced analytic solutions is not trivial. Thus, we recommend that organizations perform a total cost of ownership analysis. This should include operating costs, maintenance, annual license fees and upgrade costs, and backup and recovery costs as well as other relevant expenses.
3. **Validate and pilot test:** Whether your organization is developing or buying an advanced analytics solution, validating and testing the solution beyond the initial development is crucial. Vendors of health information technology have an incentive to overstate the value of their tools [10]. Sensitivity, specificity, positive and negative predictive value, C statistic, Hosmer-Lemeshow test, and other pertinent calibration and discrimination metrics should be scrutinized before implementation. Following validation, a pilot should be run to test the analytics solution and the clinical workflow integration strategy using representative clinicians who would use the provided information. Also, pilots offer insight into the best way to communicate new information (risk score, diagnostic, etc.) to clinicians at the point of care. In addition, piloting will help with development of a strategy for implementing the solution across the enterprise.
4. **Measure performance and evaluate:** With the potential to impact the lives of thousands of patients, developing a measurement system that facilitates the evaluation of advanced analytics solutions is essential. Measuring and reporting back to the relevant stakeholders the pertinent clinical, safety, patient satisfaction outcomes, and costs will provide oversight of the performance of the solution and its effects on patients and costs. Using short- and long-term balancing measures will help evaluate more comprehensively the overall equity and cost-benefit of the implemented solution.
5. **Invest in data scientists:** We are awash with advanced analytics solutions, but the capacity and expertise needed to develop, implement, and evaluate analytic tools is limited in many health care systems. Data scientists can help integrate the growing number of streams of data into actionable knowledge. The skillset of a data scientist is heterogenous and reflects the wide range of backgrounds of these professionals (mathematicians, statisticians, medical informaticists, epidemiologists, etc.). Simply hiring data scientists is not enough—you must also ensure that their role is clearly defined. Data scientists should be placed strategically on multidisciplinary teams with clinicians in order to give them exposure to clinical processes of care. This approach can also help clinicians become more comfortable with the knowledge generated from these advanced analytics solutions. Once they have been oriented to what is expected of them, data scientists should be given autonomy and support to be creative. After all, as Steve Jobs famously said, “it doesn’t make sense to hire smart people and tell them what to do; we hire smart people so they can tell us what to do.”

## The future ahead

There is no question that advanced analytics will become an essential solution for improving health care. Arming our clinical experts with the best advanced analytics will enhance the delivery of care by allowing them to focus on issues directly related to patient care. However, careful and objective considerations need to be made before implementing a solution. Critically evaluating any advanced analytics solution before, during, and after its implementation will ensure safe care, good outcomes, and the elimination of waste.
